# Small operator ideals formed by *s* numbers on generalized Cesáro and Orlicz sequence spaces

**DOI:** 10.1186/s13660-018-1945-y

**Published:** 2018-12-29

**Authors:** Nashat Faried, Awad A. Bakery

**Affiliations:** 1 Department of Mathematics, Faculty of Science and Arts, University of Jeddah, Khulais, Saudi Arabia; 2 Department of Mathematics, Faculty of Science, Ain Shams University, Cairo, Egypt

**Keywords:** *s*-numbers, Small operator ideal, Orlicz sequence space, Generalized Cesáro sequence space

## Abstract

In this article, we establish sufficient conditions on the generalized Cesáro and Orlicz sequence spaces $\mathbb{E}$ such that the class $S_{\mathbb{E}}$ of all bounded linear operators between arbitrary Banach spaces with its sequence of *s*-numbers belonging to $\mathbb{E}$ generates an operator ideal. The components of $S_{\mathbb{E}}$ as a pre-quasi Banach operator ideal containing finite dimensional operators as a dense subset and its completeness are proved. Some inclusion relations between the operator ideals as well as the inclusion relations for their duals are obtained. Finally, we show that the operator ideal formed by $\mathbb{E}$ and approximation numbers is small under certain conditions.

## Introduction

The operator ideals theory is gaining importance in functional analysis, since it has many applications in spectral theory, geometry of Banach spaces, eigenvalue distributions theorem, fixed point theorem, etc. Throughout this paper, by *w* we denote the space of all real sequences, $\mathbb{R}$ denotes the real numbers, $\mathbb{N}=\{0, 1, 2,\ldots\}$, and $\mathfrak{L}(U, V)$ is the space of all bounded linear operators from a normed space *U* into a normed space *V*. Some of operator ideals in the class of Banach spaces or Hilbert spaces are defined by different scalar sequence spaces. For example the ideal of compact operators is defined by the space $c_{0}$ of convergent to zero sequences and Kolmogorov numbers. Pietsch [1] examined the operator ideals formed by the classical sequence space $\ell ^{p}$ ($0< p< \infty $) and the approximation numbers. He showed that the ideal of nuclear operators and the ideal of Hilbert–Schmidt operators between Hilbert spaces are defined by $\ell ^{1}$ and $\ell ^{2}$, respectively, and the sequence of approximation numbers. In [2], the authors studied the operator ideals constructed by generalized Cesáro and Orlicz sequence spaces $\ell _{M}$ and approximation numbers. With continuity in generalization, the idea of this paper is to study a generalized class $S_{\mathbb{E}}$ by using some sequences of *s*-numbers and $\mathbb{E}$. We give sufficient conditions on Orlicz and generalized Cesáro sequence spaces $\mathbb{E}$ such that the class $S_{\mathbb{E}}$ forms an operator ideal. The completeness and denseness of its ideal components are specified. We also prove that the class $S_{\mathbb{E}}$, for any pre-modular special space of sequences (sss), is a pre-quasi Banach operator ideal which is more general than the usual classes of operator ideals. Moreover, we have obtained various inclusion relations between the operator ideals as well as the inclusion relations for their duals. Finally, we give sufficient conditions on Orlicz and generalized Cesáro sequence spaces such that the operator ideal formed by approximation numbers is small. These results are considered as a generalization for the case of $\ell ^{p}$, ($0< p<\infty $).

## Definitions and preliminaries

### Definition 2.1

([3])

An *s*-number function is a map defined on $\mathfrak{L}(U, V)$ which associates to each operator $P\in \mathfrak{L}(U, V)$ a sequence of nonnegative numbers $(s_{n}(P))_{n=0}^{\infty} $ with some properties:

- monotonicity: $\|P \|=s_{0}(P)\geq s_{1}(P)\geq s_{2}(P) \geq \cdots\geq 0$ for all $P\in \mathfrak{L}(U, V)$.
- additivity: $s_{m+n-1}(P_{1}+P_{2})\leq s_{m}(P_{1})+s_{n}(P _{2})$ for all $P_{1}, P_{2}\in \mathfrak{L}(U, V)$, *m*, $n\in \mathbb{N}$.
- property of ideal: $s_{n}(TPR)\leq \|T \| s_{n}(P) \|R \|$ for all $R\in \mathfrak{L}(U_{0}, U)$, $P\in \mathfrak{L}(U, V)$, and $T\in \mathfrak{L}(V, V_{0})$, where $U_{0}$ and $V_{0}$ are normed spaces.
- $s_{n}(\lambda P)=|\lambda |s_{n}(P)$ for every $T\in \mathfrak{L}(U, V)$, $\lambda \in \mathbb{R}$.
- rank property: If $\operatorname{rank}(P)\leq n$, then $s_{n}(P)=0$ for every $P\in \mathfrak{L}(U, V)$.
- property of norming:
  $$ s_{i}(I_{j})= \textstyle\begin{cases} 1, & \text{if }i< j; \\ 0, & \text{if }i\geq j, \end{cases} $$
  where $I_{j}$ is the identity operator on the Euclidean space $\mathbb{R} ^{j}$.

There are several examples of *s*-numbers, we mention the following:

1. The *n*th approximation number, denoted by $\alpha _{n}(P)$, is defined by $\alpha _{n}(P)=\inf \{ \|P-A \|:A\in \mathfrak{L}(U, V) \text{ and }\operatorname{rank}(A)\leq n\}$.
2. The *n*th Gel’fand number, denoted by $c_{n}(P)$, is defined by $c_{n}(P)=\alpha _{n}(J_{V} P)$, where $J_{V}$ is a metric injection from the normed space *V* to a higher space $l_{\infty }(\varLambda )$ for an adequate index set *Λ*. This number is independent of the choice of the higher space $l_{\infty }(\varLambda )$.
3. The *n*th Kolmogorov number, denoted by $d_{n}(P)$, is defined by
  $$ d_{n}(P)=\inf_{\operatorname{dim} V\leq n}\sup_{ \Vert x \Vert \leq 1}\inf _{y\in V} \Vert Px-y \Vert . $$
4. The *n*th Weyl number, denoted by $x_{n}(P)$, is defined by
  $$ x_{n}(P)=\inf \bigl\{ \alpha _{n}(P A): \|A:\ell _{2}\rightarrow U\|\leq 1\bigr\} . $$
5. The *n*th Chang number, denoted by $y_{n}(P)$, is defined by
  $$ y_{n}(P)=\inf \bigl\{ \alpha _{n}(BP): \|B:V\rightarrow \ell _{2}\|\leq 1\bigr\} . $$
6. The *n*th Hilbert number, denoted by $h_{n}(P)$, is defined by
  $$ h_{n}(P)=\sup \bigl\{ \alpha _{n}(BPA): \|B:V\rightarrow \ell _{2}\|\leq 1 \text{ and }\|A:\ell _{2}\rightarrow U\|\leq 1\bigr\} . $$

### Remark 2.2

([3])

Among all the *s*-number sequences defined above, it is easy to verify that the approximation number $\alpha _{n}(P)$ is the largest and the Hilbert number $h_{n}(P)$ is the smallest *s*-number sequence, i.e., $h_{n}(P)\leq s_{n}(P)\leq \alpha _{n}(P)$ for any bounded linear operator *P*. If *P* is compact and defined on a Hilbert space, then all the *s*-numbers coincide with the eigenvalues of $|P|$, where $|P | = (P^{*}P)^{\frac{1}{2}}$.

### Theorem 2.3

([3], p. 115)

*If* $P\in \mathfrak{L}(U, V)$, *then*

$$ h_{n}(P)\leq x_{n}(P)\leq c_{n}(P)\leq \alpha _{n}(P)\quad \textit{and} \quad h _{n}(P)\leq y_{n}(P)\leq d_{n}(P)\leq \alpha _{n}(P). $$

### Theorem 2.4

([3], p. 90)

*An* *s*-*number sequence is injective if*, *for any metric injection* $J\in \mathfrak{L}(V,V_{0})$, $s_{n}(P)=s_{n}(JP)$ *for all* $P\in \mathfrak{L}(U, V)$.

### Theorem 2.5

([3], p. 95)

*An* *s*-*number sequence is surjective if*, *for any metric surjection* $Q\in \mathfrak{L}(U_{0}, U)$, $s_{n}(P)=s_{n}(PQ)$ *for all* $P\in \mathfrak{L}(U, V)$.

### Theorem 2.6

([3], pp. 90–94)

*The Gel’fand numbers and the Weyl numbers are injective*.

### Theorem 2.7

([3], pp. 95)

*The Kolmogorov numbers and the Chang numbers are surjective*.

### Definition 2.8

A finite rank operator is a bounded linear operator whose dimension of the range space is finite.

### Definition 2.9

((Dual *s*-numbers) [4])

For each *s*-number sequence $s=(s_{n})$, a dual *s*-number function $s^{D} = (s^{D}_{n})$ is defined by

$$ s^{D}_{n}(P)=s_{n}\bigl(P^{\prime }\bigr) \quad \text{for all } P\in \mathfrak{L}(U, V), $$

where $P^{\prime }$ is the dual of *P*.

### Definition 2.10

([5], p. 152)

An *s*-number sequence is called symmetric if $s_{n}(P)\geq s_{n}(P^{\prime })$ for all $P\in \mathfrak{L}(U, V)$. If $s_{n}(P)=s_{n}(P^{\prime })$, then the *s*-number sequence is said to be completely symmetric.

Now we recall some known results related to the dual of an *s*-number sequence.

### Theorem 2.11

([5], p. 152)

*The approximation numbers are symmetric*, *i*.*e*., $\alpha _{n}(P^{\prime })\leq \alpha _{n}(P)$ *for* $P\in \mathfrak{L}(U, V)$.

### Remark 2.12

([6])

$\alpha _{n}(P^{\prime })=\alpha _{n}(P)$ for every compact operator *P*.

### Theorem 2.13

([5], p. 153)

*If* $P\in \mathfrak{L}(U, V)$, *then*

$$ c_{n}(P)=d_{n}\bigl(P^{\prime }\bigr)\quad \textit{and} \quad c_{n}\bigl(P^{\prime }\bigr)\leq d_{n}(P). $$

*In addition*, *if* *P* *is a compact operator*, *then* $c_{n}(P^{\prime })=d_{n}(P)$.

### Theorem 2.14

([3], p. 96)

*If* $P\in \mathfrak{L}(U, V)$, *then*

$$ x_{n}(P)=y_{n}\bigl(P^{\prime }\bigr)\quad \textit{and} \quad y_{n}\bigl(P^{\prime }\bigr)\leq x_{n}(P), $$

*i*.*e*., *Weyl numbers and Chang numbers are dual to each other*.

### Theorem 2.15

([5], p. 153)

*The Hilbert numbers are completely symmetric*, *i*.*e*., $h_{n}(P)=h_{n}(P^{\prime })$ *for all* $P\in \mathfrak{L}(U, V)$.

### Definition 2.16

([7, 8])

The operator ideal $\mathbb{U}:= \{ \mathbb{U}(U, V); U \text{ and } V\text{ are Banach spaces} \}$ is a subclass of linear bounded operators such that its components $\mathbb{U}(U, V)$ which are subsets of $\mathfrak{L}(U, V)$ satisfy the following conditions:

(i) $I_{K}\in \mathbb{U}$ where *K* indicates a one-dimensional Banach space, where $\mathbb{U}\subset \mathfrak{L}$.
(ii) For $P_{1}, P_{2}\in \mathbb{U}(U, V)$, then $\lambda _{1}P _{1}+\lambda _{2} P_{2}\in \mathbb{U}(U, V)$ for any scalars $\lambda _{1}$, $\lambda _{2}$.
(iii) If $P\in \mathfrak{L}(U_{0}, U)$, $T\in \mathbb{U}(U, V)$, and $R\in \mathfrak{L}(V, V_{0})$, then $RTP\in U(U_{0}, V_{0})$.

### Definition 2.17

An Orlicz function is a function $M:[0,\infty )\rightarrow [0,\infty )$ which is convex, continuous, and nondecreasing with $M(0)= 0$, $M (u)>0$ for $u>0$ and $M (u)\rightarrow \infty $, as $u\rightarrow \infty $. See [9] and [10].

### Definition 2.18

An Orlicz function *M* is said to satisfy $\Delta _{2}$-condition for every value of $u\geq 0$ if there is $k>0$ such that $M(2u)\leq k M (u)$. The $\Delta _{2}$-condition is equivalent to $M(lu)\leq k l M (u)$ for every value of $l>1$ and *u*.

Lindentrauss and Tzafriri [11] utilized the idea of an Orlicz function to define Orlicz sequence space:

$$\begin{aligned} \ell _{M}= \bigl\{ u\in \omega :\rho (\beta u)< \infty \text{ for some } \beta >0 \bigr\} \quad \text{where } \rho (u)=\sum ^{\infty }_{k=0}M\bigl( \vert u _{k} \vert \bigr), \end{aligned}$$

$(\ell _{M}, \|\cdot\|)$ is a Banach space with the Luxemburg norm:

$$\begin{aligned} \Vert u \Vert =\inf \biggl\{ \beta >0:\rho \biggl(\frac{u}{\beta }\biggr) \leq 1 \biggr\} . \end{aligned}$$

Every Orlicz sequence space contains a subspace that is isomorphic to $c_{0}$ or $\ell ^{q}$ for some $1\leq q<\infty $ ([12], Theorem 4.a.9).

Later, several classes of sequences have been introduced using Orlicz functions by Altin et al. [13], Et et al. ([14] and [15]), and Tripathy et al. [16–18].

For a sequence $q=(q_{i})$ of positive real numbers with $q_{i}\geq 1$, for all $i\in \mathbb{N}$, the generalized Cesáro sequence space is defined by

$$\begin{aligned}& \mathrm{ces}\bigl((q_{i})\bigr)= \bigl\{ u=(u_{i})\in \omega :\rho (\beta u)< \infty \text{ for some }\beta >0 \bigr\} \quad \text{and}\\& \rho (u)=\sum^{\infty }_{i=0} \biggl( \frac{\sum^{i}_{j=0} \vert u_{j} \vert }{i+1} \biggr)^{q_{i}}. \end{aligned}$$

$(\mathrm{ces}((q_{i})), \|\cdot\|)$ is a Banach space with the Luxemburg norm. If $(q_{i})$ is bounded, one can simply write

$$\begin{aligned} \mathrm{ces}\bigl((q_{i})\bigr)= \Biggl\{ u=(u_{i})\in \omega :\sum^{\infty }_{i=0} \biggl( \frac{ \sum^{i}_{j=0} \vert u_{j} \vert }{i+1} \biggr)^{q_{i}}< \infty \Biggr\} . \end{aligned}$$

Sanhan and Suantai [19] studied some geometric properties of $\mathrm{ces}((q_{i}))$.

### Definition 2.19

([2])

A class of linear sequence spaces $\mathbb{E}$ is called a special space of sequences (sss) if

1. $e_{i}\in \mathbb{E}$ for all $i\in \mathbb{N}$,
2. if $u=(u_{i})\in w$, $v=(v_{i})\in \mathbb{E}$ and $|u_{i}|\leq |v_{i}|$ for every $i\in \mathbb{N}$, then $u\in \mathbb{E}$ “i.e., $\mathbb{E}$ is solid”,
3. if $(u_{i})_{i=0}^{\infty }\in \mathbb{E}$, then $(u_{[\frac{i}{2}]})_{i=0}^{\infty }\in \mathbb{E}$, wherever $[\frac{i}{2}]$ means the integral part of $\frac{i}{2}$.

### Theorem 2.20

$\ell _{M}$ *is a* (*sss*) *if* *M* *is an Orlicz function satisfying* $\Delta _{2}$-*condition*.

### Theorem 2.21

$\mathrm{ces}((q_{i}))$ *is a* (*sss*) *if* $(q_{i})$ *is an increasing sequence*, $1 < q_{0}$ *and* $\sup q_{i}<\infty $.

### Definition 2.22

([2])

A subclass of the special space of sequences called a pre-modular (sss) if there is a function $\varrho : \mathbb{E}\rightarrow [0,\infty [$ satisfying the following conditions:

(i) $\varrho (u)\geq 0$ for each $u\in \mathbb{E}$ and $\varrho (u)=0\Leftrightarrow u=\theta $, where *θ* is the zero element of $\mathbb{E}$,
(ii) there exists $L\geq 1$ such that $\varrho (\beta u)\leq L| \beta |\varrho ( u)$ for all $u\in \mathbb{E}$ and for any scalar *β*,
(iii) for some $K\geq 1$, $\varrho (u+v)\leq K(\varrho (u)+ \varrho (v))$ for every $u, v\in \mathbb{E}$,
(iv) if $|u_{i}|\leq |v_{i}|$ for all $i\in \mathbb{N}$, then $\varrho ((u_{i}))\leq \varrho ((v_{i}))$,
(v) for some $K_{0}\geq 1$, $\varrho ((u_{i}))\leq \varrho ((u _{[\frac{i}{2}]}))\leq K_{0}\varrho ((u_{i}))$,
(vi) the set of all finite sequences is *ϱ*-dense in $\mathbb{E}$. This means that, for each $u=(u_{i})_{i=o}^{\infty }\in \mathbb{E}$ and for each $\varepsilon > 0$, there exists $s\in \mathbb{N}$ such that $\varrho ((u_{i})_{i=s} ^{\infty })<\varepsilon $,
(vii) there exists a constant $\xi >0$ such that $\varrho ( \beta , 0, 0, 0,\ldots)\geq \xi |\beta |\varrho (1, 0, 0, 0,\ldots)$ for any $\beta \in \mathbb{R}$.

From condition (ii), it is clear that *ϱ* is continuous at *θ*. We denote by $(\mathbb{E}_{\varrho }, \varrho )$ the linear space $\mathbb{E}$ equipped with the metrizable topology generated by *ϱ*.

### Example 2.23

$\ell ^{q}$ is a pre-modular (sss) if $0< q<\infty $.

### Example 2.24

$\ell _{M}$ is a pre-modular (sss) if *M* is an Orlicz function satisfying $\Delta _{2}$-condition.

### Example 2.25

$\mathrm{ces}_{q}$ is a pre-modular (sss) if $1< q<\infty $.

### Example 2.26

$\mathrm{ces}((q_{i}))$ is a pre-modular (sss) if $(q_{i})$ is an increasing sequence, $1 < q_{0}$ and $\sup q_{i}<\infty $.

### Theorem 2.27

([20])

*If* *U*, *V* *are infinite dimensional Banach spaces and* $(\mu _{i})$ *is a monotonic decreasing sequence to zero*, *then there exists a bounded linear operator* *P* *such that*

$$ \frac{1}{16}\mu _{3i}\leq \alpha _{i}(P)\leq 8\mu _{i+1}. $$

Throughout this paper, $e_{i}=\{0, 0,\ldots,1,0,\ldots\}$ where 1 appears at the $i{th}$ place for all $i\in \mathbb{N}$, the sequence $(q_{i})$ is a bounded sequence of positive numbers, and the following well-known inequality [21]: $|a_{i}+b_{i}|^{q_{i}}\leq K(|a_{i}|^{q _{i}}+|b_{i}|^{q_{i}})$, where $K=2^{h-1}$, $h=\sup_{i}q_{i}$, and $q_{i}\geq 1$ for every $i\in \mathbb{N}$ are used.

## Main results

### Notations 3.1

$$ \begin{aligned}[b] &S_{\mathbb{E}}:= \bigl\{ S_{\mathbb{E}}(U, V); U \text{ and } V \text{ are Banach spaces} \bigr\} , \quad \text{where} \\ & S_{\mathbb{E}}(U, V):= \bigl\{ P\in \mathfrak{L}(U, V):\bigl(s_{i}(P) \bigr)_{i=0} ^{\infty }\in \mathbb{E} \bigr\} . \quad \text{Also} \\ & S_{\mathbb{E}}^{\mathrm{app}}:= \bigl\{ S_{\mathbb{E}}^{\mathrm{app}}(U, V); U \text{ and } V\text{ are Banach spaces} \bigr\} , \text{where} \\ & S_{\mathbb{E}}^{\mathrm{app}}(U, V):= \bigl\{ P\in \mathfrak{L}(U, V):\bigl( \alpha _{i}(P)\bigr)_{i=0}^{\infty }\in \mathbb{E} \bigr\} . \end{aligned} $$

### Theorem 3.2

*If* $\mathbb{E}$ *is a* (*sss*), *then* $S_{\mathbb{E}}$ *is an operator ideal*.

### Proof

To show $S_{\mathbb{E}}$ is an operator ideal

(i) let $B\in F(U, V)$ and $\operatorname{rank}(B)=n$ for each $n\in \mathbb{N}$, since $e_{i}\in \mathbb{E}$ for each $i\in \mathbb{N}$ and $\mathbb{E}$ is a linear space, hence $(s_{i}(B))_{i=0}^{\infty }=( s_{0}(B), s_{1}(B),\ldots,s_{n-1}(B), 0, 0, 0,\ldots)= \sum^{n-1}_{i=0}s_{i}(B)e_{i}\in \mathbb{E}$; for that $B\in S_{\mathbb{E}}(U, V)$, which implies $F(U, V)\subseteq S_{E}(U, V)$.

(ii) Let $P_{1}, P_{2}\in S_{\mathbb{E}}(U, V)$ and $\beta _{1}, \beta _{2}\in \mathbb{R}$, then from Definition 2.19 condition (3) we get $(s_{[\frac{i}{2}]}(P_{1}))_{i=0}^{\infty }\in \mathbb{E}$ and $(s_{[\frac{i}{2}]}(P_{1}))_{i=0}^{\infty }\in \mathbb{E}$, since $i\geq 2[\frac{i}{2}]$, from the definition of *s*-numbers and $s_{i}(P)$ is a decreasing sequence, we have $s_{i}(\beta _{1}P_{1}+\beta _{2}P_{2})\leq s_{2[\frac{i}{2}]}(\beta _{1}P_{1}+\beta _{2}P_{2})\leq s_{[\frac{i}{2}]}(\beta _{1}P_{1})+s_{[ \frac{i}{2}]}(\beta _{2}P_{2})=|\beta _{1}|s_{[\frac{i}{2}]}(P_{1})+| \beta _{2}|s_{[\frac{i}{2}]}(P_{2})$ for all $i\in \mathbb{N}$. Since from Definition 2.19 condition (2) and $\mathbb{E}$ is a linear space, we have $(s_{i}(\beta _{1}P_{1}+\beta _{2}P_{2}))_{i=0}^{\infty }\in \mathbb{E}$, hence $\beta _{1}P_{1}+\beta _{2}P_{2}\in S_{\mathbb{E}}(U, V)$.

(iii) If $P\in \mathfrak{L}(U_{0}, U)$, $T\in S_{\mathbb{E}} (U, V)$, and $R\in \mathfrak{L}(V, V_{0})$, then we get $(s_{i}(P))_{i=0}^{\infty }\in \mathbb{E}$ and since $s_{i}(RTP) \leq \|R \| s_{i}(T) \|P \|$, from Definition 2.19 conditions (1) and (2) we get $(s_{i}(RTP)_{i=0}^{\infty }) \in \mathbb{E}$, then $RTP\in S_{ \mathbb{E}}(U_{0}, V_{0})$. □

### Corollary 3.3

*If* *M* *is an Orlicz function satisfying* $\Delta _{2}$-*condition*, *then* $S_{\ell _{M}}$ *is an operator ideal*.

### Corollary 3.4

$S_{\ell ^{q}}$ *is an operator ideal if* $0< q<\infty $.

### Corollary 3.5

*If* $(q_{i})$ *is a bounded increasing sequence and* $q_{0}>1$, *then* $S_{\mathrm{ces}((q_{i}))}$ *is an operator ideal*.

### Corollary 3.6

$S_{\mathrm{ces}_{q}}$ *is an operator ideal if* $1< q<\infty $.

The following question arises naturally: for which Orlicz and Cesáro sequence spaces $\mathbb{E}$, is the ideal of the finite rank operators dense in $S_{\mathbb{E}}(U, V)$?

### Theorem 3.7

$S_{\ell _{M}} (U, V)=\overline{F(U, V)}$ *if* *M* *is an Orlicz function and* *U*, *V* *are Banach spaces*.

### Proof

Define $\varrho (u)=\sum_{i=0}^{\infty }M(|u_{i}|)$ on $\ell _{M}$. First, we show, if $P\in F(U, V)$, then it belongs to $S_{\ell _{M}}(U, V)$. Since $e_{i}\in \ell _{M}$ for each $i\in \mathbb{N}$ and $\ell _{M}$ is a linear space, for each finite operator $P\in F(U, V)$, we obtain $(s_{i}(P))_{i=0}^{\infty }$ containing only finitely many terms different from zero. Currently we prove that $S_{\ell _{M}} (U,V) \subseteq \overline{F(U, V)}$, let $P\in S_{\ell _{M}}(U, V)$ we have $(s_{i}(P))_{i=0}^{\infty }\in \ell _{M}$ and since $\sum_{i=0}^{ \infty }M(s_{i}(P))<\infty $, let $\varepsilon \in (0, 1]$, hence there exists $i_{0}\in \mathbb{N}-\{0\}$ with $\sum_{i=i_{0}}^{\infty }M(s_{i}(P))<\frac{\varepsilon }{4}$. Since $s_{i}(P)$ is decreasing for each $i\in \mathbb{N}$ and *ϱ* is nondecreasing, we obtain

$$ i_{0}M\bigl(s_{2i_{0}}(P)\bigr)\leq \sum _{i=i_{0}+1}^{2i_{0}}M\bigl( s_{i}(P)\bigr)\leq \sum _{i=i_{0}}^{\infty }M\bigl(s_{i}(P)\bigr)< \frac{\varepsilon }{4}. $$

Hence, there exists $B\in F_{2i_{0}}(U, V)$ such that $\operatorname{rank}B\leq 2i _{0}$ with $M(\|P-B\|)<\frac{\varepsilon }{4i_{0}}$, and *M* is an Orlicz function, hence

$$ \begin{aligned}[b] d(P, B)&= \varrho \bigl(s_{i}(P-B) \bigr)_{i=0}^{\infty }\\ &=\sum_{i=0}^{\infty }M \bigl(s _{i}(P-B)\bigr) \\ &\quad{}\times \sum_{i=0}^{3i_{0}-1}M\bigl(s_{i}(P-B) \bigr)+\sum_{i=3i_{0}}^{\infty }M\bigl(\bigl(s _{i}(P-B)\bigr)\bigr) \\ & \leq \sum_{i=0}^{3i_{0}-1}M(\|P-B)\|)+\sum _{i=3i_{0}}^{\infty }M\bigl(\bigl(s _{i}(P-B) \bigr)\bigr) \\ & \leq 3i_{0}M\bigl( \Vert P-B \Vert \bigr)+\sum _{i=i_{0}}^{\infty }M\bigl(\bigl(s_{i+2i_{0}}(P-B)\bigr) \bigr) \\ & \leq 3i_{0}M\bigl( \Vert P-B \Vert \bigr)+\sum _{i=i_{0}}^{\infty }M\bigl(s_{i}(P)\bigr)< \varepsilon . \end{aligned} $$

 □

### Corollary 3.8

$S_{\ell ^{q}} (U,V)=\overline{F(U, V)}$ *if* $0< q<\infty $ *and* *U*, *V* *are Banach spaces*.

### Theorem 3.9

$S_{\mathrm{ces}((q_{i}))} (U,V)=\overline{F(U, V)}$ *if* $(q_{i})$ *is an increasing sequence*, $q_{0}>1$ *and* *U*, *V* *are Banach spaces*.

### Proof

We prove first that $\overline{F(U, V)}\subseteq S_{\mathrm{ces}((q_{i}))} (U,V)$. Since $e_{i} \in \mathrm{ces}((q_{i}))$ for each $i\in \mathbb{N}$ and $\mathrm{ces}((q_{i}))$ is a linear space, for each finite operator $P\in F(U, V)$, i.e., one obtains that $(s_{i}(P))_{i=0} ^{\infty }$ holds main finitely a significant unique number in relation to zero. Now we prove that $S_{\mathrm{ces}((q_{i}))}(U,V)\subseteq \overline{F(U, V)}$. Since $q_{0}>1$ and $(q_{i})$ is an increasing sequence, we have $\sum_{i=0}^{\infty }( \frac{1}{i+1})^{q_{i}}<\infty $, let $P\in S_{\mathrm{ces}((q_{i}))}(U, V)$, we get $(s_{i}(P))_{i=0}^{\infty }\in \mathrm{ces}((q_{i}))$ and since $\varrho (s _{i}(P))_{i=0}^{\infty }<\infty $, let $\varepsilon \in (0, 1)$, hence there exists $i_{0}\in \mathbb{N}-\{0\}$ such that $\varrho ((s_{i}(P))_{i=i _{0}}^{\infty })<\frac{\varepsilon }{2^{h+3}\delta C}$ for some $c\geq 1$, where $\delta =\max \{1,\sum_{i=i_{0}}^{\infty }( \frac{1}{i+1})^{q_{i}} \}$. As $s_{i}(P)$ is decreasing for every $i\in \mathbb{N}$, we have

1 $$ \sum_{i=i_{0}+1}^{2i_{0}} \biggl(\frac{ \sum_{j=0}^{i}s_{2i_{0}}(P)}{i+1} \biggr)^{q_{i}}\leq \sum_{i=i_{0}+1}^{2i_{0}} \biggl(\frac{ \sum_{j=0}^{i}s_{j}(P)}{i+1} \biggr)^{q_{i}}\leq \sum _{i=i_{0}}^{\infty } \biggl(\frac{ \sum_{j=0}^{i}s_{j}(P)}{i+1} \biggr)^{q_{i}}< \frac{\varepsilon }{2^{h+3} \delta C}. $$

Hence, there exists $B\in F_{2i_{0}}(U, V)$ such that $\operatorname{rank}B\leq 2i _{0}$ and

2 $$ \sum_{i=2i_{0}+1}^{3i_{0}} \biggl(\frac{ \sum_{j=0}^{i} \Vert P-B \Vert }{i+1} \biggr)^{q_{i}} \leq \sum_{i=i_{0}+1}^{2i_{0}} \biggl(\frac{ \sum_{j=0}^{i} \Vert P-B \Vert }{i+1} \biggr)^{q_{i}} < \frac{\varepsilon }{2^{h+3} \delta C} $$

for the bounded sequence $(q_{i})$. Then consider

3 $$ \sup_{i=i_{0}}^{\infty } \Biggl( \sum _{j=0}^{i_{0}} \Vert P-B \Vert \Biggr)^{q_{i}}< \frac{\varepsilon }{2^{2h+2} \delta }, $$

hence by setting

4 $$ \sum_{i=0}^{i_{0}} \biggl(\frac{ \sum_{j=0}^{i} \Vert P-B \Vert }{i+1} \biggr)^{q_{i}}< \frac{\varepsilon }{2^{h+3} \delta C}. $$

Since $(q_{i})$ is increasing and by using (1), (2), (3), and (4), we have

$$\begin{aligned} d(P, B)&=\varrho \bigl(s_{i}(P-B) \bigr)_{i=0}^{\infty } = \sum_{i=0}^{3i_{0}-1} \biggl(\frac{ \sum_{j=0}^{i}s_{j}(P-B)}{i+1} \biggr)^{q_{i}}+ \sum_{i=3i_{0}}^{\infty } \biggl(\frac{ \sum_{j=0}^{i}s_{j}(P-B)}{i+1} \biggr)^{q_{i}} \\ & \leq \sum_{i=0}^{3i_{0}} \biggl( \frac{ \sum_{j=0}^{n} \Vert P-B \Vert }{i+1} \biggr)^{q_{i}}+ \sum_{i=i_{0}}^{\infty } \biggl(\frac{ \sum_{j=0}^{i+2i_{0}}s_{j}(P-B)}{i+1} \biggr)^{q_{i+2i_{0}}} \\ &\leq \sum_{i=0}^{3i_{0}} \biggl( \frac{ \sum_{j=0}^{i} \Vert P-B \Vert }{i+1} \biggr)^{q_{i}}+ \sum_{i=i_{0}}^{\infty } \biggl(\frac{ \sum_{j=0}^{i+2i_{0}}s_{j}(P-B)}{i+1} \biggr)^{q_{i}} \\ &\leq \sum_{i=0}^{3i_{0}} \biggl( \frac{ \sum_{j=0}^{i} \Vert P-B \Vert }{i+1} \biggr)^{q_{i}}+ \sum_{i=i_{0}}^{\infty } \biggl(\frac{ \sum_{j=0}^{i+2i_{0}}s_{j}(P-B)}{i+1} \biggr)^{q_{i}} \\ &\leq 3 \sum_{i=0}^{i_{0}} \biggl( \frac{ \sum_{j=0}^{i} \Vert P-B \Vert }{i+1} \biggr)^{q_{i}}+ \sum_{i=i_{0}}^{\infty } \biggl(\frac{ \sum_{j=0}^{2i_{0}-1}s_{j}(P-B)+ \sum_{j=2i_{0}}^{i+2i_{0}}s_{j}(P-B)}{i+1} \biggr)^{q_{i}} \\ &\leq 3 \sum_{i=0}^{i_{0}} \biggl( \frac{ \sum_{j=0}^{i} \Vert P-B \Vert }{i+1} \biggr)^{q_{i}}\\ &\quad {}+ 2^{h-1} \Biggl[ \sum _{i=i_{0}}^{\infty } \biggl(\frac{ \sum_{j=0}^{2i_{0}-1}s_{j}(P-B)}{i+1} \biggr)^{q_{i}}+ \sum_{i=i_{0}}^{\infty } \biggl(\frac{ \sum_{j=2i_{0}}^{i+2i_{0}}s_{j}(P-B)}{i+1} \biggr)^{q_{i}} \Biggr] \\ &\leq 3 \sum_{i=0}^{i_{0}} \biggl( \frac{ \sum_{j=0}^{i} \Vert P-B \Vert }{i+1} \biggr)^{q_{i}}\\ &\quad {}+ 2^{h-1} \Biggl[ \sum _{i=i_{0}}^{\infty } \biggl(\frac{ \sum_{j=0}^{2i_{0}-1} \Vert P-B \Vert }{i+1} \biggr)^{q_{i}}+ \sum_{i=i_{0}}^{\infty } \biggl(\frac{ \sum_{j=0}^{i}s_{j+2i_{0}}(P-B)}{i+1} \biggr)^{q_{i}} \Biggr] \\ &\leq 3 \sum_{i=0}^{i_{0}} \biggl( \frac{ \sum_{j=0}^{i} \Vert P-B \Vert }{i+1} \biggr)^{q_{i}} +2^{h-1}\sup _{i=i_{0}} ^{\infty } \Biggl( \sum_{j=0}^{2i_{0}-1} \Vert P-B \Vert \Biggr)^{q_{i}} \sum_{i=i_{0}}^{\infty }(i+1)^{-q_{i}} \\ &\quad {}+2^{h-1} \sum_{i=i_{0}}^{\infty } \biggl( \frac{ \sum_{j=0}^{i}s_{j}(P)}{i+1} \biggr)^{q_{i}}\\ &< \varepsilon . \end{aligned}$$

 □

### Corollary 3.10

$S_{ces_{q}} (U,V)=\overline{F(U, V)}$ *if* $1< q<\infty $ *and* *U*, *V* *are Banach spaces*.

The following question arises naturally: for which Orlicz and generalized Cesáro sequence spaces $\mathbb{E}$, are the components of the ideal $S_{\mathbb{E}}^{\mathrm{app}}$ complete?

### Definition 3.11

A function $g:\varOmega \rightarrow [0, \infty )$ is said to be a pre-quasi norm on the ideal *Ω* if the following conditions hold:

1. for all $P\in \varOmega (U, V)$, $g(P)\geq 0$ and $g(P)=0$ if and only if $P=0$,
2. there exists a constant $L\geq 1$ such that $g(\lambda P) \leq L|\lambda |g(P)$ for all $P\in \varOmega (U, V)$ and $\lambda \in \mathbb{R}$,
3. there exists a constant $K\geq 1$ such that $g(P_{1}+P_{2}) \leq K[g(P_{1})+g(P_{2})]$ for all $P_{1},P_{2}\in \varOmega (U, V)$,
4. there exists a constant $C\geq 1$ such that if $T\in \mathfrak{L}(U_{0}, U)$, $P\in \varOmega (U, V)$, and $R\in \mathfrak{L}(V, V_{0})$, then $g(RPT)\leq C \|R \| g(P) \|T \|$, where $U_{0}$ and $V_{0}$ are normed spaces.

We state the following two theorems without proof, those can be established using standard techniques.

### Theorem 3.12

*Every quasi norm on the ideal* *Ω* *is a pre*-*quasi norm on the ideal* *Ω*.

### Theorem 3.13

*The function* $g(P)=\varrho (s_{i}(P))_{i=0}^{\infty }$ *is a pre*-*quasi norm on* $S_{\mathbb{E}_{\varrho }}$, *where* $\mathbb{E}_{\varrho }$ *is a pre*-*modular* (*sss*).

### Theorem 3.14

$(S_{\mathbb{E}_{\varrho }} , g)$ *is a pre*-*quasi Banach operator ideal if* $\mathbb{E}_{\varrho }$ *is a pre*-*modular* (*sss*).

### Proof

Since $\mathbb{E}_{\varrho }$ is a pre-modular (sss), then the function $g(P)=\varrho (s_{i}(P))_{i=0}^{\infty }$ is a pre-quasi norm on $S_{\mathbb{E}_{\varrho }}$. Let $(P_{m})$ be a Cauchy sequence in $S_{\mathbb{E}_{\varrho }}(U, V)$. Hence, by using Part (vii) of Definition 2.22 and since $\mathfrak{L}(U, V)\supseteq S_{ \mathbb{E}_{\varrho }}(U, V)$, one gets

$$ \begin{aligned}[b] g(P_{i}-P_{j})&= \varrho \bigl(\bigl(s_{n}(P_{i}-P_{j}) \bigr)_{n=0}^{\infty } \bigr)\geq \varrho \bigl(s_{0}(P_{i}-P_{j}), 0, 0, 0,\ldots \bigr)= \varrho \bigl( \Vert P_{i}-P_{j} \Vert , 0, 0, 0,\ldots \bigr) \\ & \geq \xi \Vert P_{i}-P_{j} \Vert \varrho (1, 0, 0, 0,\ldots), \end{aligned} $$

then $(P_{m})_{m\in \mathbb{N}}$ is a Cauchy sequence in $\mathfrak{L}(U, V)$. While the space $\mathfrak{L}(U, V)$ is a Banach space, so there exists $P\in \mathfrak{L}(U, V)$ such that $\lim_{m\rightarrow \infty } \|P_{m}-P \|=0$, and while $(s_{n}(P _{m}))_{n=0}^{\infty }\in \mathbb{E}$ for every $m\in \mathbb{N}$ , so, by using parts (iii) and (iv) of Definition 2.22 and that *ϱ* is continuous at *θ*, we obtain

$$ \begin{aligned}[b] g(P)&=\varrho \bigl(\bigl(s_{n}(P) \bigr)_{n=0}^{\infty } \bigr)= \varrho \bigl(\bigl(s _{n}(P-P_{m}+P_{m})\bigr)_{n=0}^{\infty } \bigr) \\ &\leq K\varrho \bigl(\bigl(s_{[\frac{n}{2}]}(P-P_{m}) \bigr)_{n=0}^{\infty }\bigr)+K \varrho \bigl(\bigl(s_{[\frac{n}{2}]}(P_{m})_{n=0}^{\infty } \bigr) \bigr) \\ &\leq K\varrho \bigl(\bigl( \Vert P_{m}-P \Vert \bigr)_{n=0}^{\infty } \bigr)+K\varrho \bigl(\bigl(s_{n}(P_{m})_{n=0}^{\infty } \bigr) \bigr)< \infty , \end{aligned} $$

we have $(s_{n}(P))_{n=0}^{\infty }\in \mathbb{E}$, then $P\in S_{ \mathbb{E}_{\varrho }}(U, V)$. □

### Corollary 3.15

$(S_{\ell _{M}},g)$ *is a pre*-*quasi Banach operator ideal if* *M* *is an Orlicz function satisfying* $\Delta _{2}$-*condition*.

### Corollary 3.16

$(S_{\ell ^{q}} ,g)$ *is a quasi Banach operator ideal if* $0< q<\infty $.

### Corollary 3.17

$(S_{\mathrm{ces}((q_{i}))} , g)$ *is a pre*-*quasi Banach operator ideal if* $(q_{i})$ *is an increasing sequence and* $q_{0}>1$.

### Corollary 3.18

$(S_{\mathrm{ces}_{q}}^{\mathrm{app}} ,g)$ *is a quasi Banach operator ideal*, $1< q<\infty $.

### Theorem 3.19

([20])

*For any infinite dimensional Banach spaces* *U*, *V* *and for any* $q>p>0$, *it is true that* $S^{\mathrm{app}}_{\ell ^{p}}(U, V)\varsubsetneqq S^{\mathrm{app}}_{\ell ^{q}}(U, V)\subsetneqq \mathfrak{L}(U, V)$.

### Theorem 3.20

*For any infinite dimensional Banach spaces* *U*, *V* *and for any* $1< p_{n}< q_{n}$ *for all* $n\in \mathbb{N}$, *it is true that* $S^{\mathrm{app}}_{\mathrm{ces}(p_{n})}(U, V)\varsubsetneqq S^{\mathrm{app}}_{\mathrm{ces}(q_{n})}(U, V) \subsetneqq \mathfrak{L}(U, V)$, *where* $p_{n}$ *and* $q_{n}$ *are monotonic decreasing sequences*.

### Proof

Let *U* and *V* be infinite dimensional Banach spaces and for any $1< p_{n}< q_{n}$ for every $n\in \mathbb{N}$, if $P\in S^{\mathrm{app}}_{\mathrm{ces}(p_{n})}(U, V)$, then $(\alpha _{n}(P))\in \mathrm{ces}(p_{n})$. Since $\mathrm{ces}(p_{n})\subset \mathrm{ces}(q_{n})$, hence $P\in S^{\mathrm{app}}_{\mathrm{ces}(q_{n})}(U, V)$. Next, if $q_{n}> p_{n}>1$ for every $n\in \mathbb{N}$ and $\mu _{n}=\frac{1}{\sqrt[p_{n}]{n+1}}$. So, by using Theorem 2.27, one can find $P\in \mathfrak{L}(U, V)$ with $\frac{1}{16\sqrt[p_{n}]{3n+1}}\leq \alpha _{n}(P) \leq \frac{8}{\sqrt[p _{n}]{n+2}}$ such that *P* does not belong to $S^{\mathrm{app}}_{\mathrm{ces}(p_{n})}(U, V)$ and $P\in S^{\mathrm{app}}_{\mathrm{ces}(q_{n})}(U, V)$. It is easy to see that $S^{\mathrm{app}}_{\mathrm{ces}(q_{n})}(U, V)\subset \mathfrak{L}(U, V)$. Next, if we take $\mu _{n}=\frac{1}{\sqrt[q_{n}]{n+1}}$. So, by using Theorem 2.27, one can find $P\in \mathfrak{L}(U, V)$ with $\frac{1}{16 \sqrt[q_{n}]{3n+1}}\leq \alpha _{n}(P) \leq \frac{8}{\sqrt[q_{n}]{n+2}} $ such that *P* does not belong to $S^{\mathrm{app}}_{\mathrm{ces}(q_{n})}(U, V)$. □

### Corollary 3.21

*For any infinite dimensional Banach spaces* *U*, *V* *and* $1< p< q<\infty $, *then* $S^{\mathrm{app}}_{\mathrm{ces}_{p}}(U, V)\varsubsetneqq S^{\mathrm{app}}_{\mathrm{ces}_{q}}(U, V) \subsetneqq \mathfrak{L}(U, V)$.

We now study some properties of the pre-quasi Banach operator ideal $S_{\mathbb{E}}$.

### Theorem 3.22

*If the* *s*-*number sequence is injective*, *then the pre*-*quasi Banach operator ideal* ($S_{\mathbb{E}_{\varrho }}$, *g*) *is injective*.

### Proof

Let $T\in \mathfrak{L}(U, V)$ and $J\in \mathfrak{L}(V, V_{0})$ be any metric injection. Suppose that $JT\in S_{\mathbb{E}_{\varrho }}(U, V_{0})$, then $\varrho (s_{n}(JT))< \infty $. Since the *s*-number sequence is injective, we have $s_{n}(JT)=s_{n}(T)$ for all $T\in \mathfrak{L}(U, V)$, $n\in \mathbb{N}$. So $\varrho (s_{n}(T))=\varrho (s_{n}(JT))<\infty $. Hence $T\in S_{\mathbb{E}_{\varrho }}(U, V)$ and clearly $g(T)=g(JT)$ holds. □

### Remark 3.23

The pre-quasi Banach operator ideal ($S^{\mathrm{Gel}}_{\mathbb{E}_{\varrho }}$, *g*) and the pre-quasi Banach operator ideal ($S^{\mathrm{Weyl}}_{\mathbb{E} _{\varrho }}$, *g*) are injective pre-quasi Banach operator ideals.

### Theorem 3.24

*If the* *s*-*number sequence is surjective*, *then the pre*-*quasi Banach operator ideal* ($S_{\mathbb{E}_{\varrho }}$, *g*) *is surjective*.

### Proof

Let $T\in \mathfrak{L}(U, V)$ and $Q\in \mathfrak{L}(U_{0}, U)$ be any metric surjection. Suppose that $TQ\in S_{\mathbb{E}_{\varrho }}(U_{0}, V)$. Then $\varrho (s_{n}(TQ))< \infty $. Since the *s*-number sequence is surjective, we have $s_{n}(TQ)=s_{n}(T)$ for all $T\in \mathfrak{L}(U, V)$, $n\in \mathbb{N}$. So $\varrho (s_{n}(T))=\varrho (s_{n}(TQ))<\infty $. Hence $T\in S_{E_{\varrho }}(U, V)$ and clearly $g(T)=g(TQ)$ holds. □

### Remark 3.25

The pre-quasi Banach operator ideal ($S^{\mathrm{Kol}}_{\mathbb{E}_{\varrho }}$, *g*) and the pre-quasi Banach operator ideal ($S^{\mathrm{Chang}}_{\mathbb{E} _{\varrho }}$, *g*) are surjective pre-quasi Banach operator ideals.

Also, we have the following inclusion relations between the pre-quasi Banach operator ideals.

### Theorem 3.26

1. $S^{\mathrm{app}}_{\mathbb{E}_{\varrho }}\subseteq S^{\mathrm{Gel}}_{ \mathbb{E}_{\varrho }}\subseteq S^{\mathrm{Weyl}}_{\mathbb{E}_{\varrho }} \subseteq S^{\mathrm{Hilb}}_{\mathbb{E}_{\varrho }}$.
2. $S^{\mathrm{app}}_{\mathbb{E}_{\varrho }}\subseteq S^{\mathrm{Kol}}_{ \mathbb{E}_{\varrho }}\subseteq S^{\mathrm{Chang}}_{\mathbb{E}_{\varrho }} \subseteq S^{\mathrm{Hilb}}_{\mathbb{E}_{\varrho }}$.

### Proof

Since $h_{n}(P)\leq x_{n}(P)\leq c_{n}(P)\leq \alpha _{n}(P)$ and $h_{n}(P)\leq y_{n}(P)\leq d_{n}(P)\leq \alpha _{n}(P)$ for every $n\in \mathbb{N}$ and *ϱ* is nondecreasing, we obtain

$$ \begin{aligned}[b] &\varrho \bigl(h_{n}(P)\bigr)\leq \varrho \bigl(x_{n}(P)\bigr)\leq \varrho \bigl(c_{n}(P) \bigr) \leq \varrho \bigl(\alpha _{n}(P)\bigr), \\ &\varrho \bigl(h_{n}(P)\bigr)\leq \varrho \bigl(y_{n}(P) \bigr)\leq \varrho \bigl(d_{n}(P)\bigr) \leq \varrho \bigl(\alpha _{n}(P)\bigr). \end{aligned} $$

Hence the result. □

We now state the dual of the operator ideal formed by different *s*-number sequences.

### Theorem 3.27

*The operator ideal* $S^{\mathrm{app}}_{\mathbb{E}_{\varrho }}$ *is symmetric and the operator ideal* $S^{\mathrm{Hilb}}_{\mathbb{E}_{\varrho }}$ *is completely symmetric*.

### Proof

Since $\alpha _{n}(P^{\prime })\leq \alpha _{n}(P)$ and $h_{n}(P^{\prime })=h_{n}(P)$ for all $P\in \mathfrak{L}(U, V)$, we have $S^{\mathrm{app}}_{\mathbb{E}_{\varrho }}\subseteq (S^{\mathrm{app}}_{\mathbb{E}_{ \varrho }})^{\prime }$ and $S^{\mathrm{Hilb}}_{\mathbb{E}_{\varrho }}=(S^{\mathrm{Hilb}}_{ \mathbb{E}_{\varrho }})^{\prime }$. □

In view of Theorem 2.13, we state the following result without proof.

### Theorem 3.28

*The operator ideal* $S^{\mathrm{Gel}}_{\mathbb{E}_{\varrho }}=(S^{\mathrm{Kol}}_{E_{ \varrho }})^{\prime }$ *and* $S^{\mathrm{Kol}}_{\mathbb{E}_{\varrho }}\subseteq (S^{\mathrm{Gel}} _{\mathbb{E}_{\varrho }})^{\prime }$. *In addition if* *T* *is a compact operator from* *U* *to* *V*, *then* $S^{\mathrm{Kol}}_{E_{\varrho }}=(S^{\mathrm{Gel}}_{E_{\varrho }})^{\prime }$.

In view of Theorem 2.14, we state the following result without proof.

### Theorem 3.29

*The operator ideal* $S^{\mathrm{Weyl}}_{\mathbb{E}_{\varrho }}=(S^{\mathrm{Chang}}_{ \mathbb{E}_{\varrho }})^{\prime }$ *and* $S^{\mathrm{Chang}}_{\mathbb{E}_{\varrho }}=(S ^{\mathrm{Weyl}}_{\mathbb{E}_{\varrho }})^{\prime }$.

### Theorem 3.30

*If* $(q_{i})$ *is an increasing sequence and* $q_{0}>1$, *then the pre*-*quasi Banach operator ideal* $S^{\mathrm{app}}_{\mathrm{ces}(q_{i})}$ *is small*.

### Proof

Since $(q_{i})$ is an increasing sequence and $q_{0}>1$, take $\lambda = (\sum_{i=0}^{\infty }\frac{1}{(i+1)^{q _{i}}} )^{\frac{1}{h}}$. Then $(S^{\mathrm{app}}_{\mathrm{ces}(q_{i})}, g)$, where $g(P)=\frac{1}{\lambda } (\sum_{i=0}^{\infty }(\frac{\sum_{j=0} ^{i}\alpha _{j}(P)}{(i+1)})^{q_{i}} )^{\frac{1}{h}}$ is a pre-quasi Banach operator ideal. Let *U* and *V* be any two Banach spaces. Suppose that $S^{\mathrm{app}}_{\mathrm{ces}(q_{i})}(U, V)=\mathfrak{L}(U, V)$, then there exists a constant $C>0$ such that $g(P)\leq C\|P\|$ for all $P\in \mathfrak{L}(U, V)$. Assume that *U* and *V* are infinite dimensional Banach spaces. Hence by Dvoretzky’s theorem [5] for $m\in \mathbb{N}$, we have quotient spaces $U/N_{m}$ and subspaces $M_{m}$ of *V* which can be mapped onto $\ell _{2}^{m}$ by isomorphisms $H_{m}$ and $A_{m}$ such that $\|H_{m}\|\|H_{m}^{-1}\|\leq 2$ and $\|A_{m}\|\|A_{m}^{-1}\|\leq 2$. Let $I_{m}$ be the identity map on $\ell _{2}^{m}$, $Q_{m}$ be the quotient map from *U* onto $U/N_{m}$, and $J_{m}$ be the natural embedding map from $M_{m}$ into *V*. Let $u_{n}$ be the Bernstein numbers [4], then

5 $$ \begin{aligned}[b] 1&= u_{n}(I_{m})=u_{n} \bigl(A_{m}A_{m}^{-1}I_{m}H_{m}H_{m}^{-1} \bigr) \\ & \leq \Vert A_{m} \Vert u_{n}\bigl(A_{m}^{-1}I_{m}H_{m} \bigr) \bigl\Vert H_{m}^{-1} \bigr\Vert \\ & = \Vert A_{m} \Vert u_{n}\bigl(J_{m}A_{m}^{-1}I_{m}H_{m} \bigr) \bigl\Vert H_{m}^{-1} \bigr\Vert \\ & \leq \Vert A_{m} \Vert d_{n}\bigl(J_{m}A_{m}^{-1}I_{m}H_{m} \bigr) \bigl\Vert H_{m}^{-1} \bigr\Vert \\ & = \Vert A_{m} \Vert d_{n}\bigl(J_{m}A_{m}^{-1}I_{m}H_{m}Q_{m} \bigr) \bigl\Vert H_{m}^{-1} \bigr\Vert \\ & \leq \Vert A_{m} \Vert \alpha _{n} \bigl(J_{m}A_{m}^{-1}I_{m}H_{m}Q_{m} \bigr) \bigl\Vert H_{m}^{-1} \bigr\Vert \end{aligned} $$

for $1\leq i\leq m$. Now

$$ \begin{aligned}[b] &\sum_{j=0}^{i}(1) \leq \sum_{j=0}^{i} \Vert A_{m} \Vert \alpha _{j}\bigl(J_{m}A_{m} ^{-1}I_{m}H_{m}Q_{m}\bigr) \bigl\Vert H_{m}^{-1} \bigr\Vert \\ &\quad \Rightarrow\quad \frac{1}{i+1}(i+1)\leq \Vert A_{m} \Vert \Biggl( \frac{1}{i+1}\sum_{j=0}^{i}\alpha _{j}\bigl(J_{m}A_{m}^{-1}I_{m}H_{m}Q_{m} \bigr)\Biggr) \bigl\Vert H_{m}^{-1} \bigr\Vert \\ &\quad \Rightarrow \quad 1\leq \bigl( \Vert A_{m} \Vert \bigl\Vert H_{m}^{-1} \bigr\Vert \bigr)^{q_{i}}\Biggl(\frac{1}{i+1}\sum _{j=0}^{i} \alpha _{j}\bigl(J_{m}A_{m}^{-1}I_{m}H_{m}Q_{m} \bigr)\Biggr)^{q_{i}}. \end{aligned} $$

Therefore,

$$ \begin{aligned}[b] &\Biggl(\sum_{i=0}^{m}(1) \Biggr)^{\frac{1}{h}}\leq L \Vert A_{m} \Vert \bigl\Vert H_{m}^{-1} \bigr\Vert \Biggl[\sum _{i=0}^{m}\Biggl(\frac{1}{i+1}\sum _{j=0}^{i}\alpha _{j}\bigl(J_{m}A_{m}^{-1}I_{m}H _{m}Q_{m}\bigr)\Biggr)^{q_{i}}\Biggr]^{\frac{1}{h}} \\ &\quad \Rightarrow \quad \frac{1}{\lambda }(m+1)^{\frac{1}{h}}\leq L \Vert A_{m} \Vert \bigl\Vert H_{m}^{-1} \bigr\Vert \frac{1}{ \lambda } \Biggl[\sum_{i=0}^{m}\Biggl( \frac{1}{i+1}\sum_{j=0}^{i}\alpha _{j}\bigl(J_{m}A _{m}^{-1}I_{m}H_{m}Q_{m} \bigr)\Biggr)^{q_{i}}\Biggr]^{\frac{1}{h}} \\ &\quad \Rightarrow\quad \frac{1}{\lambda }(m+1)^{\frac{1}{h}}\leq L \Vert A_{m} \Vert \bigl\Vert H_{m}^{-1} \bigr\Vert g\bigl(J _{m}A_{m}^{-1}I_{m}H_{m}Q_{m} \bigr), \\ &\hphantom{\quad \Rightarrow\quad \ }\frac{1}{\lambda }(m+1)^{\frac{1}{h}}\leq LC \Vert A_{m} \Vert \bigl\Vert H_{m}^{-1} \bigr\Vert \bigl\Vert J_{m}A_{m}^{-1}I_{m}H_{m}Q_{m} \bigr\Vert , \\ &\hphantom{\quad \Rightarrow\quad \ }\frac{1}{\lambda }(m+1)^{\frac{1}{h}}\leq LC \Vert A_{m} \Vert \bigl\Vert H_{m}^{-1} \bigr\Vert \bigl\Vert J_{m}A_{m}^{-1} \bigr\Vert \Vert I_{m} \Vert \Vert H_{m}Q_{m} \Vert \\ &\hphantom{\quad \Rightarrow\quad \ \frac{1}{\lambda }(m+1)^{\frac{1}{h}}}=LC \Vert A_{m} \Vert \bigl\Vert H_{m}^{-1} \bigr\Vert \bigl\Vert A_{m}^{-1} \bigr\Vert \Vert I_{m} \Vert \Vert H_{m} \Vert , \\ &\hphantom{\quad \Rightarrow\quad \ }\frac{1}{\lambda }(m+1)^{\frac{1}{h}}\leq 4LC \end{aligned} $$

for some $L\geq 1$. Thus we arrive at a contradiction since *m* is arbitrary. Thus *U* and *V* both cannot be infinite dimensional when $S^{\mathrm{app}}_{\mathrm{ces}(q_{i})}(U, V)= \mathfrak{L}(U, V)$. Hence the result. □

### Theorem 3.31

*If* $(q_{i})$ *is increasing and* $q_{0}>1$, *then the pre*-*quasi Banach operator ideal* $S^{\mathrm{Kol}}_{\mathrm{ces}(q_{i})}$ *is small*.

### Corollary 3.32

*If* $1< q<\infty $, *then the quasi Banach operator ideal* $S^{\mathrm{app}}_{\mathrm{ces} _{q}}$ *is small*.

### Corollary 3.33

*If* $1< q<\infty $, *then the quasi Banach operator ideal* $S^{\mathrm{Kol}}_{\mathrm{ces} _{q}}$ *is small*.

### Theorem 3.34

*If* *M* *is an Orlicz function satisfying* $\Delta _{2}$-*condition*, *then the pre*-*quasi Banach operator ideal* $S^{\mathrm{app}}_{\ell _{M}}$ *is small*.

### Proof

Since *M* is an Orlicz function satisfying $\Delta _{2}$-condition, then $(S^{\mathrm{app}}_{\ell _{M}}, g)$, where $g(P)=\sum_{i=0}^{\infty } M(\alpha _{i}(P))$ is a pre-quasi Banach operator ideal. Let *U* and *V* be any two Banach spaces. Suppose that $S^{\mathrm{app}}_{\ell _{M}} (U, V)= \mathfrak{L}(U, V)$, then there exists a constant $C>0$ such that $g(P)\leq C\|P\| $ for all $P \in \mathfrak{L}(U, V)$. Assume that *U* and *V* are infinite dimensional Banach spaces. By using inequality (5) and since *M* is an Orlicz function satisfying $\Delta _{2}$-condition, one obtains

$$ \begin{aligned}[b] &M(1)\leq L \Vert A_{m} \Vert M \bigl(\alpha _{i}\bigl(J_{m}A_{m}^{-1}I_{m}H_{m}Q_{m} \bigr)\bigr) \bigl\Vert H _{m}^{-1} \bigr\Vert \\ &\quad \Rightarrow \quad \sum_{i=0}^{m}1\leq L \Vert A_{m} \Vert \bigl\Vert H_{m}^{-1} \bigr\Vert \sum_{i=0}^{m}M\bigl(\alpha _{i}\bigl(J_{m}A_{m}^{-1}I_{m}H_{m}Q_{m} \bigr)\bigr) \\ &\quad \Rightarrow\quad (m+1)\leq L \Vert A_{m} \Vert \bigl\Vert H_{m}^{-1} \bigr\Vert g\bigl(J_{m}A_{m}^{-1}I_{m}H_{m}Q_{m} \bigr), \\ &\hphantom{\quad \Rightarrow\quad\ }(m+1)\leq LC \Vert A_{m} \Vert \bigl\Vert H_{m}^{-1} \bigr\Vert \bigl\Vert J_{m}A_{m}^{-1}I_{m}H_{m}Q_{m} \bigr\Vert , \\ &\hphantom{\quad \Rightarrow\quad\ }(m+1)\leq LC \Vert A_{m} \Vert \bigl\Vert H_{m}^{-1} \bigr\Vert \bigl\Vert J_{m}A_{m}^{-1} \bigr\Vert \Vert I_{m} \Vert \Vert H _{m}Q_{m} \Vert \\ &\hphantom{\quad \Rightarrow\quad (m+1)}=LC \Vert A_{m} \Vert \bigl\Vert H_{m}^{-1} \bigr\Vert \bigl\Vert A_{m}^{-1} \bigr\Vert \Vert I_{m} \Vert \Vert H_{m} \Vert , \\ &\hphantom{\quad \Rightarrow\quad\ }(m+1)\leq 4LC \end{aligned} $$

for some $L\geq 1$. Thus we arrive at a contradiction since *m* is arbitrary. Thus *U* and *V* both cannot be infinite dimensional when $S^{\mathrm{app}}_{\ell _{M}}(U, V)= \mathfrak{L}(U, V)$. Hence the result. □

### Corollary 3.35

([22])

*If* $0< p<\infty $, *then the quasi Banach operator ideal* $S^{\mathrm{app}}_{\ell ^{p}}$ *is small*.

### Corollary 3.36

*If* $0< p<\infty $, *then the quasi Banach operator ideal* $S^{\mathrm{Kol}}_{\ell ^{p}}$ *is small*.
